# Identification of MiRNA–Disease Associations Based on Information of Multi-Module and Meta-Path

**DOI:** 10.3390/molecules27144443

**Published:** 2022-07-11

**Authors:** Zihao Li, Xing Huang, Yakun Shi, Xiaoyong Zou, Zhanchao Li, Zong Dai

**Affiliations:** 1 School of Biomedical Engineering, Sun Yat-sen University, Shenzhen 518107, China; lizh377@mail2.sysu.edu.cn (Z.L.); huangx48@mail2.sysu.edu.cn (X.H.); shiyk6@mail2.sysu.edu.cn (Y.S.); 2 School of Chemistry, Sun Yat-sen University, Guangzhou 510275, China; 3 School of Chemistry and Chemical Engineering, Guangdong Pharmaceutical University, Guangzhou 510006, China

**Keywords:** MiRNA–disease association, graph neural network, meta-path

## Abstract

Cumulative research reveals that microRNAs (miRNAs) are involved in many critical biological processes including cell proliferation, differentiation and apoptosis. It is of great significance to figure out the associations between miRNAs and human diseases that are the basis for finding biomarkers for diagnosis and targets for treatment. To overcome the time-consuming and labor-intensive problems faced by traditional experiments, a computational method was developed to identify potential associations between miRNAs and diseases based on the graph attention network (GAT) with different meta-path mode and support vector (SVM). Firstly, we constructed a multi-module heterogeneous network based on the meta-path and learned the latent features of different modules by GAT. Secondly, we found the average of the latent features with weight to obtain a final node representation. Finally, we characterized miRNA–disease-association pairs with the node representation and trained an SVM to recognize potential associations. Based on the five-fold cross-validation and benchmark datasets, the proposed method achieved an area under the precision–recall curve (AUPR) of 0.9379 and an area under the receiver–operating characteristic curve (AUC) of 0.9472. The results demonstrate that our method has an outstanding practical application performance and can provide a reference for the discovery of new biomarkers and therapeutic targets.

## 1. Introduction

MicroRNA (miRNA), with a length between 18 and 24 nucleotides, is one of the types of non-coding RNAs in cells. Previously, miRNA was considered as a useless clip of human gene and even once called ‘junk gene’ because it could not encode protein [1]. However, more and more research studies show that miRNA is able to regulate the gene expression affecting some essential biological processes, such as proliferation, division, growth and apoptosis of the cell [2,3,4]. It commonly binds with messenger RNA (mRNA) at the three prime untranslated region (3′UTR) to achieve the transcription repression or degradation of the mRNA target [5]. Therefore, a high or low level of miRNA can lead to chaotic protein synthesis, which may destroy normal metabolism and cause dysfunction, further inviting diseases [6]. In addition, some studies have also shown that miRNA serving as an epigenetic regulator of gene expression goes hand in hand with human diseases [7,8,9,10]. Therefore, to identify the association between miRNAs and diseases is helpful for understanding the pathogenesis of disease. Moreover, miRNA can serve as a promising biomarker for diagnosis or a target for treatment [11]. Nevertheless, traditional biological experiments, limited by high cost, and being laborious and time consuming, are prone to failure to find all the relations between miRNA and disease. With the development of database and biotechnology, the massive accumulation of biological data enables researchers to extract potential information and further adopt it to identify miRNA–disease association (MDA). Protein is an essential component of all cells and tissues in the body. Protein-related information has been utilized in plenty of bioinformatics studies, such as protein interaction and drug–protein interaction [12,13,14].

Up to now, a large number of computational methods have been developed to recognize MDA. These methods, roughly classified into three categories of similarity-based methods, network-based methods and machine learning based methods, are all based on the hypothesis that miRNAs with similar function tend to be associated with diseases with similar phenotypes. For similarity-based methods, throughout the development of the computational method for MDA, Jiang et al. [15] used a computation method instead of a traditional experiment to find unknown MDA. The work made use of the genes related to miRNA and hypergeometric distributions to calculate the miRNA similarity score and find the perspective neighbor miRNA by ranking the score; however, it only focused on the direct neighbor miRNA and neglected the undirect ones. Chen et al. [16] tried to integrate various heterogeneous biological datasets and calculate the within-score and between-score to rank the indefinite MDA. Pasquier et al. [17] utilized diverse information to construct miRNA and disease vector and find MDA by vector similarity. The network-based method predicts MDA by implementing random walk and other propagation algorithms in miRNA and disease network. Chen et al. [18] constructed the miRNA functional similarity network (MFSN) and implemented the random walk algorithm on it to obtain the score of candidate miRNAs. Xuan et al. [19] divided the miRNA into two categories, labeled and unlabeled, and also carried out a random walk on the MFSN, which enabled the prior information to improve the current information. However, these methods can only be used for miRNAs that have similar function to other miRNAs. To extend the prediction, some researchers have integrated a diverse biological dataset. You et al. [20] constructed a heterogeneous graph and developed a path-based method adopting a depth-first search algorithm to surmise MDA. Chen et al. [21] designed a method which implements random walk on the miRNA–miRNA and disease–disease network constructed by Laplacian score of graphs, respectively. The development of machine learning and deep learning breathes new life into the fields of healthcare and bioinformatics, such as disease prediction, sleep monitoring and medical image processing [22,23,24]. In addition, many methods based on machine learning and deep learning have been proposed to distinguish associations between miRNA and diseases. Jiang et al. [25] employed the miRNA and disease similarity score as a feature vector and randomly selected some unobserved MDA as negative samples to classify by support vector machine. Zhao et al. [26] attempted to integrate several decision trees to obtain the score with a respective weight, forming a strong classifier, which achieved an adaptive boosting improvement for prediction. Li et al. [27] utilized a graph convolution network to learn the latent feature of miRNA and disease. Subsequently, they acquired MDA by neural inductive matrix completion. Xuan et al. [28] constructed a dual convolutional neural network framework to learn the global and local representation for the subsequent prediction. Ji et al. [29] gained the miRNA and disease representation, respectively, by minimizing the squared losses between the value of cosine distance and the score of the function similarity, and then adopted the auto encoder to predict the probability of MDA.

Recently, graph neural network, depending on its ability to fuse the feature of node and graph topological structure, has been introduced into bioinformatics [13,30,31,32,33]. What is more, the introduction of meta-path is able to enrich the semantic information of the network and provide the extra structure information for uncovering the complexity of the network. As mentioned above, a protein whose chaos in synthesis may cause diseases plays an essential role in life activity as well as being regulated by miRNA. Thus, the integration of protein, miRNA and disease information may be able to significantly improve the prediction performance.

Inspired by graph neural networks such as graph convolutional network (GCN) [34], graph attention network (GAT) [35] and heterogenous graph attention network [36], a novel method is proposed for predicting miRNA–disease association. In the current approach, multi-module meta-path along with graph attention network is employed to extract the network topology features of miRNAs and diseases, and support vector machine (SVM) is used as classifier to identify the potential MDA (MMGAN-SVM). Finally, five-fold cross-validation is conducted to evaluate the prediction performance and the case studies with lymphoma, liver neoplasms and lung neoplasms are performed to demonstrate the practical application performance.

Overall, the main contributions of this work are as follows:

1. Protein information and meta-path strategy were utilized to construct the multi-module, which can enrich the information of miRNAs and diseases.
2. The topological and semantic information can be better learned by Graph attention network and attention mechanism.
3. A reliable negative sample selection strategy was utilized to overcome the imbalance between positive and negative samples.

## 2. Results

### 2.1. Dimension Optimization of Node Representation

**Figure 1:**
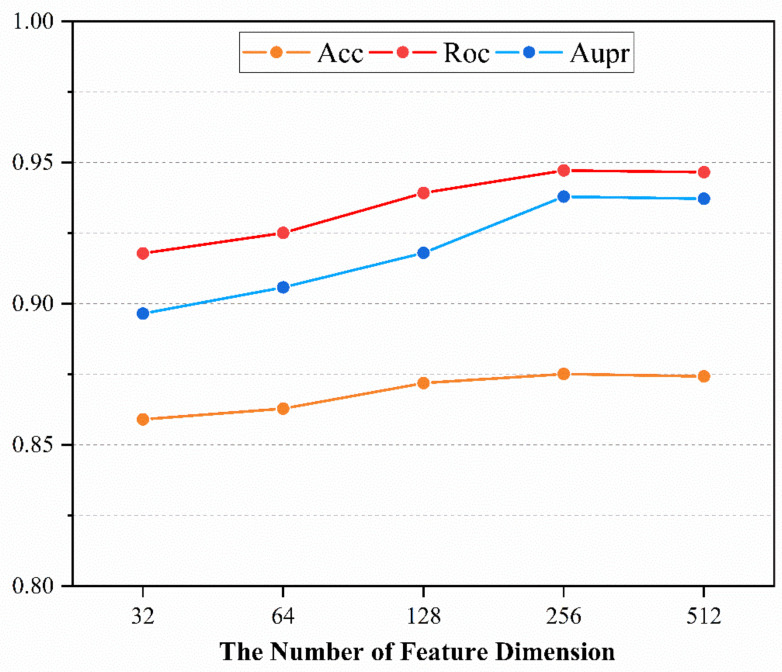
The main performance under different dimensions.

**Figure 2:**
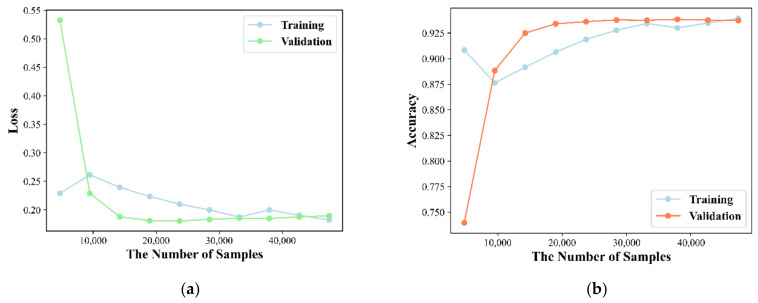
The learning curve of our method. (**a**) Training and validation accuracy graph; (**b**) Training and validation error graph.

The node representation implies the complex information in latent feature space, and its dimensionality affects the predictive performance of the model. A low number of dimensions may lead to the loss of information, while a high number of dimensions will lead to the introduction of noise and time consuming for calculation. Thus, discovery of the optimized dimension of the node representation is attempted based on the 5-CV through changing dimension in the range of (32, 64, 128, 256, 512). The experiment is repeated 10 times for each dimension. Here, Acc, Roc and Aupr are utilized to evaluate the effect of dimension on model performance and statistical average results are shown in Figure 1. The Acc of each dimension is 0.8591, 0.8628, 0.8719, 0.8751 and 0.8700 and its standard deviation (std) is 0.0030, 0.0038, 0.0033, 0.0023 and 0.0040. The Roc of each dimension is 0.9178, 0.9251, 0.9392, 0.9472 and 0.9448 and its std is 0.0031, 0.0054, 0.0030, 0.0016 and 0.0034. The Aupr of each dimension is 0.8965, 0.9058, 0.9180, 0.9379 and 0.9401 and its std is 0.0052, 0.0054, 0.0063, 0.0042 and 0.0043. We can conclude that higher dimensionality tends to be better performance. However, a high feature vector can lead to a huge computational burden and long model training time. Therefore, the optimal feature dimension for node representation is set to 256. The learning curve of our model is shown in Figure 2 and the result illustrates that the model has been trained in an optimal state.

### 2.2. Classifier Optimization

**Figure 3:**
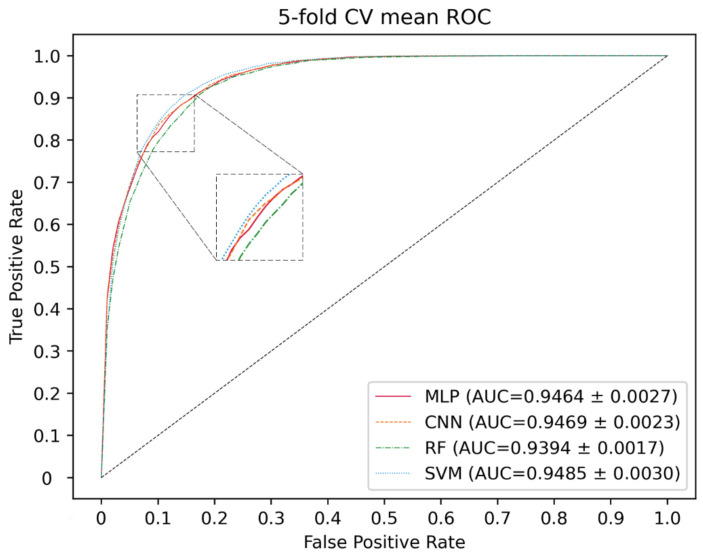
ROC curves for different classifier.

**Figure 4:**
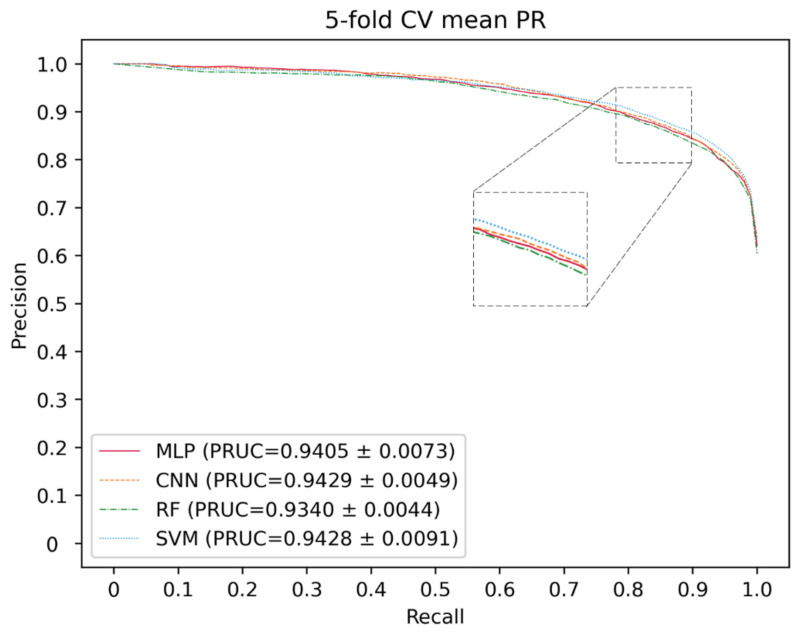
PR curves for different classifier.

**Figure 5:**
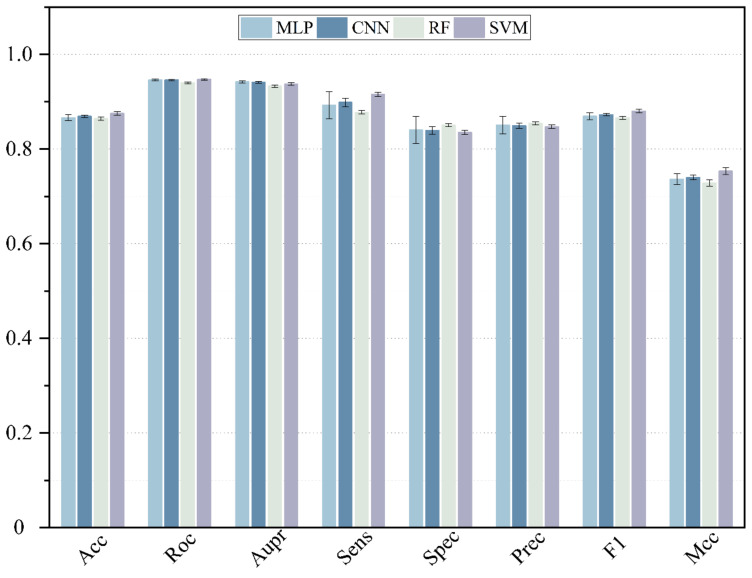
The performance comparison of different classifier.

Here, deep neural networks (DNNs), such as multi-layer perceptron (MLP), convolutional neural networks (CNN) and the traditional machine learning method, including SVM and random forest (RF), are utilized to construct a model. The 5-CV is conducted with a different model 10 times in the same condition as well as with the optimal parameter, and the result is shown in the Figure 3, Figure 4 and Figure 5 and in Table 1. In the 5-CV experiment, SVM shows the best performance in Auc and Aupr. Although the evaluation measures of SVM in 10 repetitive experiments are a little better than those of other models, SVM performs a lower std than other models. In conclusion, SVM shows the better performance in the majority of evaluation measures.

### 2.3. Comparison with Other Methods

**Figure 6:**
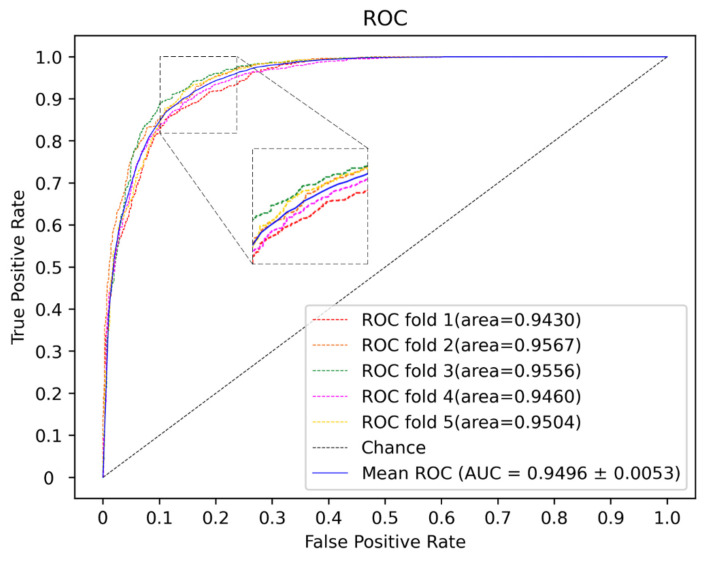
The ROC curves of our method.

**Figure 7:**
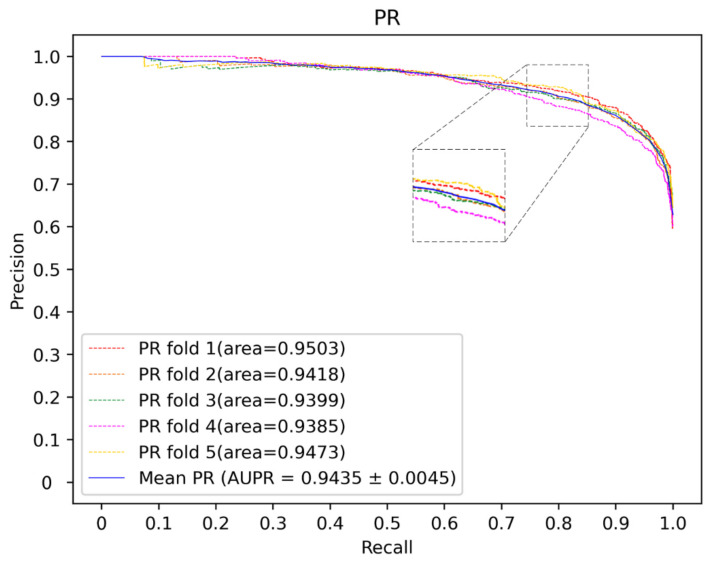
The PR curves of our method.

**Figure 8:**
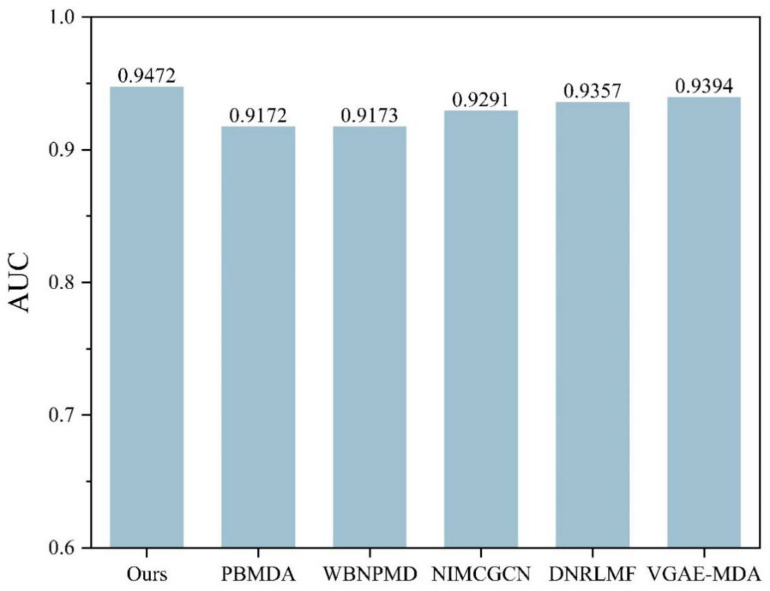
Performance comparison of different methods in 5-CV.

To further demonstrate the performance of the current method, a comparison is performed with some state of art methods including PBMDA [20], WBNPMD [37], NIMCGCN [27], DNRLMF-MDA [38] and VGAE-MDA [39]. PBMDA is a path-based method which aims at eliminating weak interactions. WBNPMD predicted the MDA by the bipartite network projection with weight. NIMCGCN is a matrix completion-based method which learns the feature by GCN. DNRLMF-MDA is a matrix factorization-based method and it utilized dynamic neighborhood regularization to improve performance. VGAE-MDA adopted variational graph auto-encoders to integrate the score from well-trained two subgraphs. Based on the benchmark dataset, the best results of 5-CV from our model are shown in Figure 6 and Figure 7. The average of Acc, Roc, Aupr and F1 measured ten times in the experiment are 0.8753, 0.9472, 0.9374 and 0.8801 with the std 0.0036, 0.0015, 0.0030 and 0.0034, respectively. The five-fold cross-validation results of the existing methods are shown in Figure 8. The AUCs of PBMDA, WBNPMD, NIMCGCN, DNRLMF-MDA and VGAE-MDA are 0.9172, 0.9173, 0.9291, 0.9357 and 0.9394, respectively. In our method, the features of miRNAs and diseases are not only enriched by extra information of the protein but also integrated with the structure semantic information of MDA. In addition, SVM can show great performances in nonlinear classification tasks. Due to these strategies, the result also illustrated correspondingly that our method presented an outstanding performance.

### 2.4. Proportion of Negative Sample

**Figure 9:**
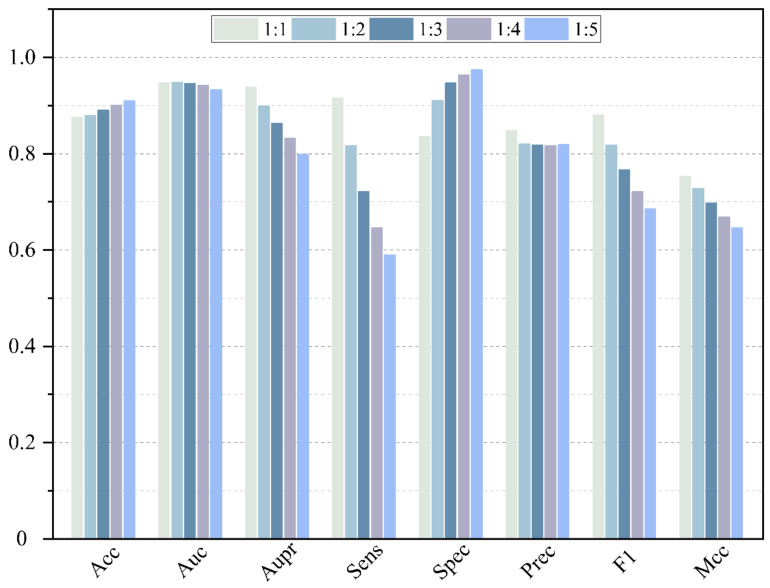
Performance comparison of different ratio of positive and negative samples.

In fact, the number of negative samples is much larger than the number of positive samples. Therefore, the impact of the ratio of positive and negative samples on the performance of the model is further investigated. A negative sample with different ratios of 1:1, 1:2, 1:3, 1:4 and 1:5 is randomly selected to conduct the 5-CV, and the result is shown in Figure 9 and listed in Table 2. As we can see, some evaluation measures are affected significantly by the unbalance between positive and negative samples, because these evaluation measures are sensitive to the ratio between positive and negative samples. With the increase in negative samples, the value of Aupr, Sens, F1 and Mcc slowly descends. A greater number of negative samples involved in the training procedure makes it easier for the model to identify the negative samples. Thus, the value of Acc increases along with the growth of ratios. The values of Auc and Aupr fluctuate within a controllable range. However, aiming at digging potential MDA, it is necessary for the model to obtain high sensitivity. Thus, to display the best performance of our model, the proportion of negative and positive samples is set as 1:1.

### 2.5. Reliability of Negative Sample

**Figure 10:**
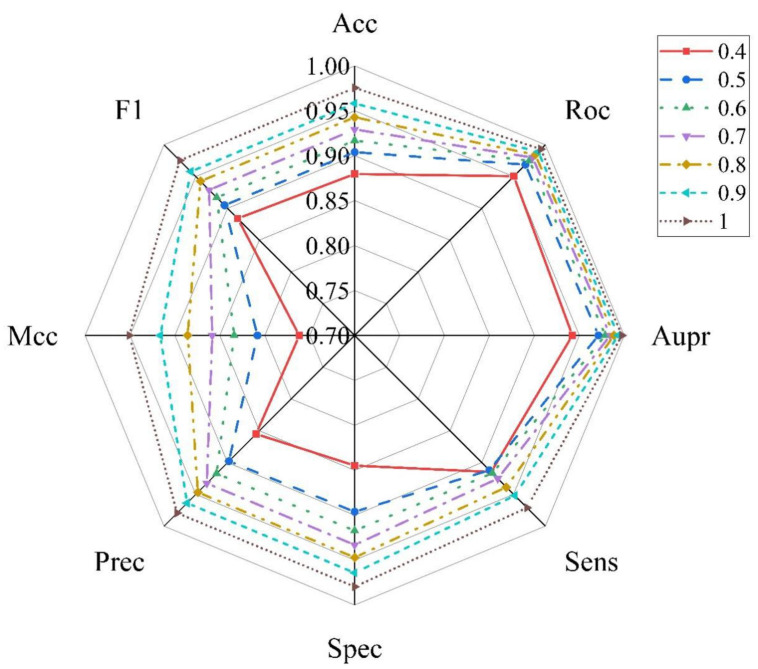
Performance comparison of different thresholds of negative samples.

At present, there is no database dedicated to collecting miRNA–disease non-association pairs because these pairs cannot provide more information to promote the mechanisms’ research and drug discovery. To overcome this problem, a random matching method is utilized to construct negative samples; however, it may contain false negative samples. Therefore, the influence of negative sample reliability on model performance is further studied. At first, we calculated the mean values of all dimensions for all the positive samples to form a cluster vector. Then, we obtained the average Euclidean distance (AED) by calculating Euclidean distance between each negative sample and the cluster vector. Depending on different threshold of AED, the original negative sample set was able to be refined and shrunk. The AED threshold was set in the range of (0.4AED, 0.5AED, 0.6AED, 0.7AED, 0.8AED, 0.9AED 1.0AED) and the negative samples whose Euclidean distance was lower than the threshold were removed to obtain different negative sample datasets. Then, negative sample with the same ratios as positive samples were randomly selected from the dataset for the training set in each of the threshold experiments. The results of different threshold are shown in Figure 10. The values of Acc, Auc, Aupr, Sens, Spec, Precision, F1 and Mcc are located in the range of (0.8798–0.9755), (0.9506–0.9932), (0.9423–0.9975), (0.9148–0.9713), (0.8448–0.9798), (0.8551–0.9795), (0.7616–0.9510) and (0.8839–0.9753), respectively. In addition, with the increase in threshold, the selected negative sample is further away from the cluster vector, and there is a degree of improvement for all the evaluation measure. Thus, the strategy of reliable negative sample selection makes a positive difference on the model.

### 2.6. Case Studies

To illustrate the practical application performance of our model, the case studies are implemented over the three common human diseases: liver neoplasm, lung neoplasm and leukemia. Specifically, the MDA information of each case study is erased during the model training and the prediction score is acquired for all the miRNA candidates. Here, the ratio of positive and negative samples is set as 1:1 and the strategy of reliable negative samples selection is utilized in training procedure. According to the prediction scores, these identified potential disease-related miRNAs are ranked in descending order. For the three diseases, the recognized top 30 miRNAs and the corresponding scores are listed in Table 3, Table 4 and Table 5, respectively. Meanwhile, these results are validated by the databases of HMDD V3.0 and dbDEMC. The latter is a database recording the expression profiles of cancer-related miRNA and the published literature [40].

Development of liver neoplasm, which has the highest mortality rate in the East Asia region, is contributed to by genetic and epigenetic factors [41]. There are two principal subtype of liver cancer, hepatocellular carcinoma (HCC) and cholangiocarcinoma, and the former is the main type happening to the case in [42]. All the top thirty miRNAs predicted can be confirmed by HMDD V3.0 or dbDEMC. In addition, some researchers reported that the over-expression of miR-221/222 is responsible for the multifocality of HCC, and the over-expression if miR-155 occurs after the cancer recurrence [43,44]. All of those miRNAs appear in the Top thirty predicted results.

Lung neoplasm is a common tumor with the highest morbidity, after breast neoplasm, worldwide, and it can be divided into two categories: small cell lung carcinoma and non-small cell lung carcinoma [45]. As listed in Table 4, all miRNAs can be validated by HMDD V3.0 or dbDEMC. Fan et al. reported the expression of miR-20a and miR-15b to be evidence to distinguish the case from healthy individuals [46]. In addition, miR-223 and miR-145 in plasma can be considered as potential biomarkers for early diagnosis [47].

Leukemia is recognized as a progressive malignant disease and is divided into four main types: acute leukemia, chronic leukemia, myelogenous leukemia and lymphocytic leukemia [48]. Twenty-nine of the top thirty predicted miRNAs in Table 5 can be validated by HMDD V3.0 or dbDEMC. Only one predicted result of miR-200b without recorded in database; however, it is revealed to promote the cell proliferation and invasion in leukemia [49]. MiR-200b acts as an oncogenic regulator in human lung cancer. The proliferation, invasion and apoptosis of leukemia cells can be controlled by miR-200b through its regulatory of NOTCH1 signaling pathway. In addition, the inactivation of miR-155 and miR-29 contribute in leukemia and the expression decrement of miR-223 can be used to distinguish the case from a healthy individual [50,51].

## 3. Discussion

As the epigenetic controller, miRNAs are involved in gene expression and cellular signaling pathways, which makes a difference in cell propagation, division, growth and apoptosis leading. With these functions, miRNAs are considered to play a critical role in the initiation and progression of human diseases as well as being the promising biomarker or therapeutic target to help with the early diagnosis and treatments. Hence, it is meaningful to discover the potential related miRNAs for a disease. In this study, a model is proposed for feature extraction and to build a model classification. The results have been compared with the state-of-the-art methods in 5-CV and the case studies showed that our method has a great performance. The comparisons of our method and other methods were performed, and advantages and drawbacks are listed in Table 6. The methods of PBMDA and WBNPMD obtained AUC of 0.9172 and 0.9173, respectively, because neither complex network was created, nor weighted edges adopted. Construction of the complex network contribute to enriching potential information of networks and adoption of a weighted edges strategy brings known microRNA disease associations into sharper focus. On the contrary, the methods of NIMCGCN, DNRLMF and VGAE-MDA with AUC of 0.9291, 0.9357 and 0.9394 not only constructed a complex network but also adopted diverse strategy to improve the prediction performance, such as neural inductive matrix completion (NIMCGCN), dynamic regularized weight (DNRLMF) and variational Bayesian inference (VGAE-MDA). Our method obtained the highest AUC of 0.9472, because the complex network was constructed and the weighted edge of microRNA disease association was considered among different modules. In addition, the weighted parameters can be adaptively learned by loss function. However, the unbalance sample problem should be investigated for all methods. The outstanding performance of our method stems from three factors. First, the information of protein is introduced to enrich the feature of miRNAs and diseases, and a composite module based on meta-path is constructed. Second, the latent feature incorporating information of the node and topological structure are extracted by node aggregation in different meta-paths and modules with an attention mechanism. Third, SVM is able to complete the non-linear classification task well on the feature extracted. In the future, much more information, such as miRNAs expression profiles, miRNA sequences and drugs, will be taken into account to improve the MDAs prediction performance. In addition, more efficient feature extraction algorithms will be a novel direction.

## 4. Materials and Methods

The experiment-verified miRNA–disease associations were retrieved from the Human microRNA Disease Database (HMDD) [52]. In this work, HMDD V2.0 was adopted as the benchmark dataset with 5430 human miRNA–disease associations incorporating 495 miRNAs and 383 diseases after deduplication and normalization. For convenience, these associations are described as an adjacent matrix $A\;\in {\;\{0,\;1\}}^{m\;\times \;n}$, in which *m* and *n* are the number of miRNAs and diseases, respectively. If an miRNA *i* is associated with a disease *j*, the value of $A_{i,j}$ is 1, and 0 vice versa. In addition, the miRNA–protein associations were collected from the miRTarBase database and the disease–protein associations from Comparative Toxicogenomics Database (CTD) [53,54].

### 4.1. Integration Similarity Calculation and Multi-Module Construction

#### 4.1.1. MiRNA Integration Similarity

MiRNA integration similarity was composed of miRNA functional similarity (MFS) and Gaussian interaction profile kernel similarity [55]. The calculation of MFS was defined according to the previous work, which was based on the assumption that the function of two miRNAs are more similar if the number of the common disease associated with them is greater [56,57]. The score of MFS was defined as FS(i, j), i.e., the similarity score between miRNA i and miRNA j. In addition, to supplement the missing entries of MFS, Gaussian interaction profile kernel similarity mGS(i, j) was adopted and defined as Equation (1):

(1) $$\mathrm{mGS}\left(i,\;j\right)=\exp\left(-\delta_{m}\|M\left(m_{i}\right)-\;M{\left(m_{j}\right)\|}^{2}\right)$$

where ${M(m}_{i})$ and ${M(m}_{j})$ indicate the ith and jth row of the adjacent matrix A, respectively. δ_m_ represents the kernel bandwidth parameter, and is illustrated as Equation (2):

(2) $$\delta_{m}=\frac{1}{m}\overset{m}{\underset{i=1}{\sum }}{\left\|M_{i}\right\|}^{2}$$

where m is the number of miRNAs. Finally, miRNA integration similarity is described as Equation (3):

(3) $$\mathrm{MS}\left(m_{i}{,m}_{j}\right)=\left\{\begin{array}{c} {\mathrm{FS}(m}_{i}{,\;m}_{j}{)\;\mathrm{If}\;\mathrm{there}\;\mathrm{is}\;a\;\mathrm{function}\;\mathrm{similarity}\;\mathrm{between}\;m}_{i}{\;\mathrm{and}\;m}_{j}\\ \mathrm{mGS}\left(m_{i}{,\;m}_{j}\right)\;\;\;\;\;\;\mathrm{otherwise} \end{array}\right.$$

#### 4.1.2. Disease Integration Similarity

Disease integration similarity constitutes disease semantic similarity and Gaussian interaction profile kernel similarity. The entry of diseases in the National Library of Medicine (http://www.ncbi.nlm.nih.gov/ (accessed on 5 November 2021)) describes the relationship among different disease, which can be used to construct a hierarchical directed acyclic graph (DAG). According to the definition by Wang et al. [56], semantic contribution of a disease d is calculated as Equation (4):

(4) $${\mathrm{DC}}_{d}\left(i\right)=\left\{\begin{array}{c} \;1\;\;\;\;\;\;\mathrm{if}\;i=d\;\\ \max\left\{{\sigma \;*\;\mathrm{DC}}_{d}\left(\;d^{\prime }\right)|\;d^{\prime }\in \;\mathrm{children}\;\mathrm{of}\;d\right\}\;\mathrm{if}\;i\;\neq \;d \end{array}\right.$$

where σ is a semantic contribution decay factor, and is maintained it as the same as the previous work that was set as 0.5 [56]. The semantic value of the disease $d_{i}$, ${\mathrm{DV}(d}_{i})$ is defined as Equation (5):

(5) $$\mathrm{DV}\left(d_{i}\right)=\underset{k\;\in \;D\left(d_{i}\right)}{\sum }{\mathrm{DC}}_{d_{i}}\left(k\right)$$

where $D\left(d_{i}\right)$ is the node set of disease $d_{i}$ and its ancestor. The semantic similarity score between disease $d_{i}$ and $d_{j}$ can be calculated as Equation (6):

(6) $$\mathrm{SS}\left(d_{i}{,\;d}_{j}\right)=\frac{\sum_{k\;\in \;D\left(d_{i}\right)\;\cap \;D\left(d_{j}\right)}\left({\mathrm{DC}}_{d_{i}}\left(k\right){+\mathrm{DC}}_{d_{j}}\left(k\right)\right)}{\mathrm{DV}\left(d_{i}\right)+\mathrm{DV}\left(d_{j}\right)}$$

Finally, the disease semantic similarity combined with Gaussian kernel similarity is defined as Equation (7):

(7) $$\mathrm{DS}\left(d_{i}{,d}_{j}\right)=\left\{\begin{array}{c} {\mathrm{SS}(d}_{i}{,\;d}_{j}{)\;\mathrm{if}\;\mathrm{there}\;\mathrm{is}\;a\;\mathrm{semantic}\;\mathrm{similarity}\;\mathrm{between}\;d}_{i}{\;\mathrm{and}\;d}_{j}\\ \mathrm{dGS}\left(d_{i}{,\;d}_{j}\right)\;\;\;\;\;\;\mathrm{otherwise} \end{array}\right.$$

where dGS is the Gaussian kernel similarity of disease and defined as Equation (8), its formulation is similar with Equation (8).

(8) $$\mathrm{dGS}\left(i,\;j\right)=\exp\left(-\delta_{m}∥\mathrm{Ds}\left(d_{i}\right)-\;\mathrm{Ds}{\left(d_{j}\right)∥}^{2}\right)$$

where Ds($d_{i}$) and Ds($d_{j}$) indicates the ith and jth column of the adjacent matrix A, respectively.

#### 4.1.3. Multi Module Construction

**Figure 11:**
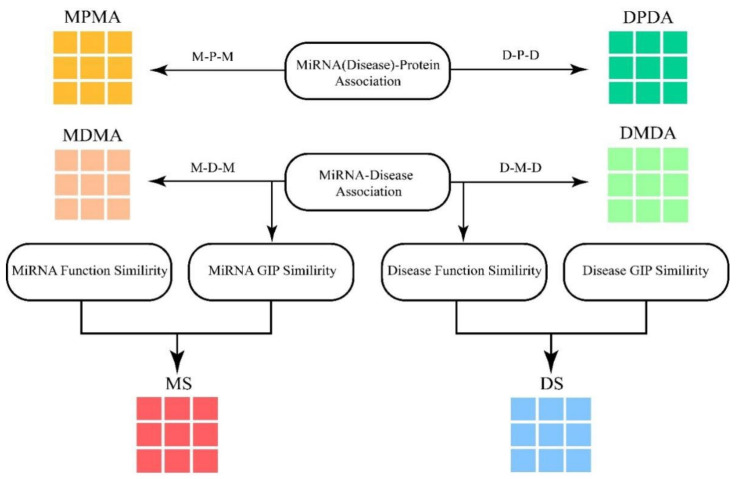
The construction of module. MPMA and MDMA are miRNA adjacent matrices based on proteins and diseases respectively. DPDA and DMDA are disease adjacent matrices based on proteins and diseases respectively. MS and DS are the similarity matrices of miRNAs and diseases respectively.

Meta-path is explained as a path in form of ${P=N}_{1}\overset{r_{1}}{\to }N_{2}\overset{r_{2}}{\to }\ldots \overset{r_{m-2}}{\to }N_{m-1}\overset{r_{m-1}}{\to }N_{m}$ (simplified as $N_{1}N_{2}{\ldots N}_{m-1}N_{m}$), which illustrates that the starting node N_1 is able to reach one of the destination nodes connected by a composite relation R = r_1 r_2..r_(m– 2) r_(m – 1) [58]. Based on the meta-path, various significance can be received from the relation between two of the identical type nodes. Thus, miRNA–protein association and disease–protein association were introduced to enrich the information of the miRNA–miRNA and disease–disease association (MMA and DDA) network. Another four association matrices MDMA (MMA based on disease), MPMA (MMA based on protein), DMDA (DDA based on miRNA) and DPDA (DDA based on protein) are shown in Figure 11.

Take MDMA for example, the value of MDMA(i, j) is 1 when miRNA i can reach miRNA j through a disease d, and it is 0 vice versa. To increase the density of MDA to about 3%, the heterogeneous graph is constructed in term of multi-module as Equations (9)–(11):

(9) $$G_{1}=\left[\begin{array}{cc} \mathrm{MS} & A\\ A^{T} & \mathrm{DS} \end{array}\right]$$

(10) $$G_{2}=\left[\begin{array}{cc} \mathrm{MDMA} & A\\ A^{T} & \mathrm{DMDA} \end{array}\right]$$

(11) $$G_{3\;}=\left[\begin{array}{cc} \mathrm{MPMA} & A\\ A^{T} & \mathrm{DPDA} \end{array}\right]$$

where the $A^{T}$ is the transposition matrix of A.

### 4.2. Information Aggregation

#### 4.2.1. Node Feature Linear Transformation and Aggregation

The original feature of miRNA and disease had to be projected into the same latent feature space, because their original feature represented two different feature spaces. The latent feature of nodes could be obtained by leveraging the transformation matrix to carry on linear transformation. Specifically, each type of node adopts a respective transformation matrix. For the node $i\;\in {\;N}_{c}$ of type c, the latent feature $h_{i}\;\in {\;R}^{d^{\prime }}$of it could be obtained by using Equation (12):

(12) $$h_{i}{=W}_{c}\;\cdot {\;x}_{c}^{i}$$

where $W_{c}\;\in {\;R}^{d^{\prime }\times \;n}$ is the transformation matrix of type c and $x_{c}^{i}\;\in {\;R}^{n}$ is the original feature of node i.

GAT was able to aggregate the information of neighboring nodes for the central node by of assigning learnable weight, which finally obtained the node representation by fusing the information of the network topological structure and node feature. Specifically, GAT adopted the SoftMax function to calculate the attention score of each node, and then continually updated the information of the central node by aggregating that of neighboring nodes based on their respective attention score. For each graph G constructed by meta-path, the importance $e_{\mathrm{ij}}^{G}$ contributed by neighbor node j to central node i was defined by Equation (13):

(13) $$e_{\mathrm{ij}}^{G}=\mathrm{LeakyReLU}\left(\omega^{G}\;\cdot \;\left[h_{i}{\;||\;h}_{j}\right]\right)$$

where $\omega^{G}\;\in {\;R}^{2\;d^{\prime }}$ is the attention parameter vector for graph G and || represents the concatenation operation. SoftMax function was utilized to normalize the importance of all nodes in order to obtain the final attention score $\alpha_{\mathrm{ij}}$ which was defined by Equation (14):

(14) $$\alpha_{\mathrm{ij}}^{G}=\mathrm{SoftMax}\left(e_{\mathrm{ij}}^{G}\right)=\frac{\exp\left(e_{\mathrm{ij}}^{G}\right)}{\sum_{k\;\in {\;N}_{i}^{G}\;}\exp\left(e_{\mathrm{ik}}^{G}\right)}$$

where k indicates the neighbor node of i in the graph G.

#### 4.2.2. Module Aggregation

Based on the attention score, the information of node i was able to aggregate that of its neighbor nodes and eventually obtain the node representation $z_{i}^{G}$ of graph G and defined as Equation (15):

(15) $$z_{i}^{G}=\sigma \left(\underset{j\;\in {\;N}_{i}^{G}}{\sum }\alpha_{\mathrm{ij}}^{G}\;\cdot {\;h}_{j}\;\right)$$

where σ(∙) represents the nonlinear activation function, and sigmoid function was used in the current study.

For the sake of the stability and low variance, multi-head attention mechanism was introduced to improve the learning process of attention score. Specifically, the aggregation was repeated for K times and the formulation (15) can be revised by Equation (16):

(16) $$z_{i}^{G}{=||}_{k=1}^{K}\;\sigma \left(\underset{j\;\in N_{i}^{G}}{\sum }\alpha_{\mathrm{ij}}^{G}\;\cdot {\;h}_{j}\right)$$

In addition, due to different meta-paths, every node obtained more than one representation in various semantic significances. To figure out which meta-path was more essential, an attention mechanism could be also adopted among the representations obtained by different meta-paths. We denoted that different meta-path mode as $G^{1}{,\;G}^{2}{,\;\ldots ,\;G}^{n}$ and the corresponding node representation as $z_{i}^{G_{1}}{,\;z}_{i}^{G_{2}}{,\;\ldots ,\;z}_{i}^{G_{n}}$. Then, an attention mechanism was used to fuse and average all of the representations with their respective weight, defined by Equations (17)–(19).

(17) $$w_{G_{k}}=\frac{1}{\left|v_{c}\right|}\;\underset{i\;\in {\;v}_{c}}{\sum }\lambda^{T}\;\cdot \tan h\left(W_{c}\;\cdot {\;z}_{i}^{G^{k}}+\varepsilon \right)$$

(18) $$\beta_{G_{k}}=\mathrm{SoftMax}\left(w_{G_{k}}\right)=\frac{\exp\left(w_{G_{k}}\right)}{\sum_{j\;\in \;\left\{G^{1}{,\;G}^{2}{,\;\ldots ,\;G}^{n}\right\}}\exp\left(w_{G_{j}}\right)}$$

(19) $$z_{i}^{\;}=\overset{\left\{G^{1}{,\;G}^{2}{,\;\ldots ,\;G}^{n}\right\}}{\underset{k=1}{\sum }}\beta_{G_{k}}\;\cdot {\;z}_{i}^{G_{k}}$$

where $W_{c}\;\in {\;R}^{{D\times d}^{\prime }}$ and $\varepsilon \;\in {\;R}^{D}$ are the weight matrix and bias vector. $v_{c}$ is a set of neighbor nodes in the same meta-path mode and $\lambda^{T}\;\epsilon {\;R}^{D}$ is the attention vector of all meta-paths for node type c. $\beta_{G_{k}}$ indicates the final attention score after normalizing the importance contribution of a meta-path and $z_{i}^{\prime }$ is the final node representation.

#### 4.2.3. Training and Prediction

Inspired by some matrix factorization or completion method, the latent feature of miRNA and disease was obtained by pre-training [59,60,61,62]. Specifically, the final node representation was used to reconstruct MDA by an inner product operation and the reconstruction error was reduced through minimizing the cross-entropy loss function defined by Equation (20):

(20) $$L\left(a,\;a^{\prime }\right)=-\left[\mathrm{alog}a^{\prime }+\left(1\;-\;a\right)\log\left(1\;-\;a^{\prime }\right)\right]$$

where a and $\;a^{\prime }$ are the original MDA and reconstruction MDA, respectively.

According to minimizing the loss function, the parameter mentioned above is constantly trained. When the well-trained latent feature of miRNAs and disease was acquired, the form of the miRNA–disease pair was concatenated as the input for SVM, which is a binary classifier with a significant accuracy and robustness in sparce and noise data. With the kernel function, it was also able to implement the non-linear classification and cater to the data complexity of miRNA and disease. After the prediction of SVM, the miRNA–disease pair association score can be obtained to indicate the association probability of the miRNA–disease pair.

### 4.3. Model Experiment and Evaluation

In this work, five-fold cross validation (5-CV) was utilized to evaluate the prediction performance of the model. The known MDA was considered as the positive sample and the stochastically selected identical amount of unobserved MDA as the negative sample. Positive and negative samples were combined into a dataset, which was randomly divided into five equal-sized subsets. Each subset was used as the test set in turn, and the remaining subsets were utilized as a training set. To reduce the variance cause by randomness, the procedure was repeated 10 times. To evaluate the performance of the model, several evaluation measures were taken into account, including accuracy (Acc), precision (Pre), specificity (Spe), sensitivity (Sens, also called as recall), F1-mesure(F1), Matthews correlation coefficient (Mcc), the areas under receiver operating characteristic (ROC) curve (AUC) and areas under precision-recall (PR) curve (AUPR). The Acc, Pre, recall and F1 were calculated by Equations (21)–(26):

(21) $$\mathrm{Acc}=\frac{\mathrm{TP}+\mathrm{TN}}{\mathrm{TP}+\mathrm{TN}+\mathrm{FP}+\mathrm{FN}}$$

(22) $$\mathrm{Pre}=\frac{\mathrm{TP}}{\mathrm{TP}+\mathrm{FP}}$$

(23) $$\mathrm{Spe}=\frac{\mathrm{TN}}{\mathrm{TP}+\mathrm{FN}}$$

(24) $$\mathrm{Recall}\left(\mathrm{Sens}\right)=\frac{\mathrm{TP}}{\mathrm{TP}+\mathrm{FN}}$$

(25) $$F1=\frac{2*\mathrm{Pre}*\mathrm{Recall}}{\mathrm{Pre}+\mathrm{Recall}}$$

(26) $$\mathrm{Mcc}=\frac{\mathrm{TP}\;\times \;\mathrm{TN}\;-\;\mathrm{FP}\;\times \;\mathrm{FN}}{\sqrt{\left(\mathrm{TP}+\mathrm{FN}\right)\;\times \;\left(\mathrm{TN}+\mathrm{FP}\right)\;\times \;\left(\mathrm{TP}+\mathrm{FP}\right)\;\times \;\left(\mathrm{TN}+\mathrm{FP}\right)}}$$

where the TP, TN, FP and FN represent the number of true positives, true negatives, false positives and false negatives, respectively.

For the part of feature extraction, we pretrained it for 4000 epochs and employed an Adam optimizer with a learning rate 0.001. In addition, the other hyper parameters only affected the dimension of node representation, and the dimension was set in the range of (32, 64, 128, 256, 512). For the part of prediction with SVM, the penalty factor C was set as 150 and the radial basis function was used as a kernel function. In addition, the architecture and parameters of the model are listed in Table 7.

**Figure 12:**
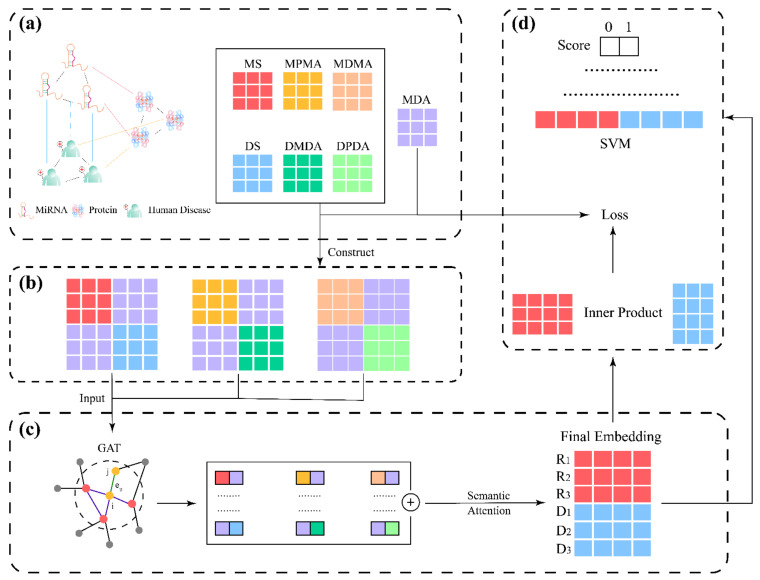
The flowchart of our method. (**a**) Construction of networks; (**b**) Construction of multi-module; (**c**) Feature extraction; (**d**) Model training and prediction.

The framework of MMGAN-SVM is illustrated in Figure 12. First of all, as shown in Figure 12a, known miRNA–disease association was coordinated with Gaussian interaction profile kernel to calculate the miRNA (disease) integrated similarity (MS/DS). The miRNA–miRNA (disease–disease) relation network was built by adopting meta-path with the help of MDA, miRNA–protein association and disease–protein association. Second, as shown in Figure 12b, with the preparation above, combination matrix constructed the multi-module to be the input of the model. Then, as shown in Figure 12c,d, the latent feature of miRNA (disease) was acquired by model concatenate in the form of an miRNA–disease pair, which later served as the input of SVM for MDA prediction.

## 5. Conclusions

In this study, extra protein information and meta-path were introduced to construct a multi-module, and GAT was utilized to learn the latent feature of the node in every module. Then, with the attention mechanism, the topological and semantic information of nodes could be aggregated adaptively by their different neighboring nodes and modules. With abundant information of latent features, the latter classification task conducted by SVM obtained a great performance in MDA prediction. In addition, the impact of different ratios of positive and negative samples on the model was explored. To some extent, the unbalance between negative and positive samples actually made some influences on the model. Thus, the strategy of reliable negative sample selection was adopted to reduce the impact of sample unbalance. In addition, the results showed that the performance of prediction can be improved by selecting negative samples within a certain threshold. In conclusion, we propose a new avenue for research to discovery potential biomarker and treatment for diseases.

## Figures and Tables

**Table 1:** The performance comparison of different classifier.

	Acc	Auc	Aupr	Sens	Spec	Prec	F1	Mcc
MLP	0.8661	0.9460	0.9420	0.8924	0.8398	0.8504	0.8693	0.7364
CNN	0.8689	0.9458	0.9411	0.8984	0.8393	0.8490	0.8725	0.7399
RF	0.8639	0.9398	0.9327	0.8776	0.8502	0.8542	0.8657	0.7281
SVM	0.8752	0.9470	0.9374	0.9156	0.83491	0.8473	0.8801	0.7531

**Table 2:** Performance comparison of different ratio of positive and negative samples.

Ratio	Acc	Auc	Aupr	Sens	Spec	Prec	F1	Mcc
1:1	0.8753	0.9470	0.9375	0.9157	0.8349	0.8473	0.8801	0.7531
1:2	0.8790	0.9481	0.8989	0.8168	0.9101	0.8199	0.8182	0.7277
1:3	0.8901	0.9460	0.8634	0.7210	0.9464	0.8177	0.7662	0.6971
1:4	0.9002	0.9422	0.8321	0.6461	0.9637	0.8167	0.7213	0.6684
1:5	0.9098	0.9325	0.7984	0.5898	0.9738	0.8186	0.6854	0.6461

**Table 3:** Top-30 Predicted Associations of Liver Neoplasms.

Rank	Score	miRNA	Evidence
1	0.9557	hsa-miR-21	HMDD3.0, dbDEMC, PMID: 31037150
2	0.9540	hsa-miR-155	dbDEMC, PMID: 29565484
3	0.9477	hsa-miR-146a	HMDD3.0, dbDEMC, PMID: 29133238
4	0.9345	hsa-miR-29a	HMDD3.0, dbDEMC, PMID: 33891266
5	0.9326	hsa-miR-16	HMDD3.0, dbDEMC, PMID: 30657555
6	0.9323	hsa-miR-29b	dbDEMC, PMID: 34184070
7	0.9309	hsa-miR-125b	HMDD3.0 dbDEMC, PMID: 32609900
8	0.9301	hsa-miR-15a	dbDEMC, PMID: 31099097
9	0.9266	hsa-miR-1	dbDEMC, PMID: 31846694
10	0.9242	hsa-miR-221	HMDD3.0, dbDEMC, PMID: 31069760
11	0.9220	hsa-miR-34a	HMDD3.0, dbDEMC, PMID: 32778238
12	0.9203	hsa-miR-17	dbDEMC, PMID: 32206115
13	0.9195	hsa-miR-20a	dbDEMC, PMID: 32206115
14	0.9184	hsa-miR-199a	HMDD3.0, dbDEMC, PMID: 31144384
15	0.9183	hsa-miR-133a	dbDEMC, PMID: 30086463
16	0.9150	hsa-miR-19b	dbDEMC, PMID: 29889802
17	0.9147	hsa-miR-29c	HMDD3.0 dbDEMC, PMID: 30718452
18	0.9141	hsa-miR-223	HMDD3.0, dbDEMC, PMID: 32233593
19	0.9139	hsa-miR-222	HMDD3.0, dbDEMC, PMID: 34273068
20	0.9101	hsa-miR-150	dbDEMC, PMID: 25549355
21	0.9043	hsa-miR-92a	dbDEMC, PMID: 32587378
22	0.9040	hsa-miR-18a	dbDEMC, PMID: 34221105
23	0.9015	hsa-miR-145	dbDEMC, PMID: 29658584
24	0.9011	hsa-miR-106b	dbDEMC, PMID: 29975452
25	0.9009	hsa-miR-181a	dbDEMC, PMID: 25058462
26	0.9006	hsa-miR-19a	dbDEMC, PMID: 27012708
27	0.8999	hsa-miR-210	HMDD3.0, dbDEMC, PMID: 27666683
28	0.8978	hsa-miR-31	HMDD3.0, dbDEMC, PMID: 25797269
29	0.8957	hsa-miR-122	HMDD3.0, dbDEMC, PMID: 25537773
30	0.8941	hsa-miR-142	HMDD3.0, dbDEMC, PMID: 30092578

**Table 4:** Top-30 Predicted Associations of Lung Neoplasms.

Rank	Score	miRNA	Evidence
1	0.9690	hsa-miR-21	HMDD3.0, dbDEMC, PMID: 30736829
2	0.9675	hsa-miR-155	HMDD3.0, dbDEMC, PMID:32447486
3	0.9673	hsa-miR-122	HMDD3.0, dbDEMC, PMID: 26604787
4	0.9672	hsa-miR-15a	HMDD3.0, dbDEMC, PMID: 33059020
5	0.9671	hsa-miR-29a	HMDD3.0, dbDEMC, PMID: 33250420
6	0.9670	hsa-miR-16	HMDD3.0, dbDEMC, PMID: 31379227
7	0.9660	hsa-miR-29b	HMDD3.0, dbDEMC, PMID: 31813135
8	0.9647	hsa-miR-133a	HMDD3.0, dbDEMC, PMID: 33074595
9	0.9630	hsa-miR-1	HMDD3.0, dbDEMC, PMID: 34139980
10	0.9626	hsa-miR-15b	dbDEMC, PMID: 32220063
11	0.9617	hsa-miR-199a	HMDD3.0, dbDEMC, PMID: 28363780
12	0.9608	hsa-miR-146a	HMDD3.0, dbDEMC, PMID: 29127520
13	0.9602	hsa-miR-29c	HMDD3.0, dbDEMC, PMID: 29512752
14	0.9598	hsa-miR-26a	HMDD3.0, dbDEMC, PMID: 33407724
15	0.9588	hsa-miR-126	HMDD3.0, dbDEMC, PMID: 34107168
16	0.9586	hsa-miR-192	HMDD3.0, dbDEMC, PMID: 29571988
17	0.9581	hsa-miR-30b	HMDD3.0, dbDEMC, PMID: 33779882
18	0.9578	hsa-miR-106b	dbDEMC, PMID: 34351868
19	0.9575	hsa-miR-19b	HMDD3.0, dbDEMC, PMID: 29455644
20	0.9569	hsa-miR-150	HMDD3.0, dbDEMC, PMID: 24456795
21	0.9575	hsa-miR-23a	HMDD3.0, dbDEMC, PMID: 28436951
22	0.9567	hsa-miR-196a	HMDD3.0, dbDEMC, PMID: 33775710
23	0.9561	hsa-miR-19a	HMDD3.0, dbDEMC, PMID: 28364280
24	0.9558	hsa-miR-23b	dbDEMC, PMID: 32495614
25	0.9556	hsa-miR-206	HMDD3.0, dbDEMC, PMID: 26919096
26	0.9555	hsa-miR-26b	HMDD3.0, dbDEMC, PMID: 26744864
27	0.9552	hsa-miR-223	HMDD3.0, dbDEMC, PMID: 29615147
28	0.9547	hsa-miR-195	HMDD3.0, dbDEMC, PMID: 32406336
29	0.9544	hsa-miR-222	HMDD3.0, dbDEMC, PMID: 32588752
30	0.9539	hsa-miR-34a	HMDD3.0, dbDEMC, PMID: 30700696

**Table 5:** Top 30 Predicted Associations of Leukemia.

Rank	Score	miRNA	Evidence
1	0.9819	hsa-miR-21	HMDD3.0, dbDEMC, PMID: 32911844
2	0.9804	hsa-miR-155	HMDD3.0, dbDEMC, PMID: 33357126
3	0.9723	hsa-miR-146a	HMDD3.0, dbDEMC, PMID: 32798394
4	0.9643	hsa-miR-17	HMDD3.0, dbDEMC, PMID: 35536524
5	0.9632	hsa-miR-29a	HMDD3.0, dbDEMC, PMID: 31870103
6	0.9631	hsa-miR-125b	HMDD3.0, dbDEMC, PMID: 27637078
7	0.9630	hsa-miR-34a	HMDD3.0, dbDEMC, PMID: 27424989
8	0.9629	hsa-miR-20a	HMDD3.0, dbDEMC, PMID: 34587164
9	0.9622	hsa-miR-16	HMDD3.0, dbDEMC, PMID: 28599250
10	0.9606	hsa-miR-221	HMDD3.0, dbDEMC, PMID: 29172404
11	0.9605	hsa-miR-29b	dbDEMC, PMID: 29435107
12	0.9568	hsa-miR-92a	HMDD3.0, dbDEMC, PMID: 31870103
13	0.9556	hsa-miR-145	HMDD3.0, dbDEMC, PMID: 32538049
14	0.9552	hsa-miR-126	HMDD3.0, dbDEMC, PMID: 34686664
15	0.9546	hsa-miR-1	dbDEMC, PMID: 28042875
16	0.9543	hsa-miR-15a	HMDD3.0, dbDEMC, PMID: 24026141
17	0.9532	hsa-miR-19b	HMDD3.0, dbDEMC, PMID: 29032147
18	0.9520	hsa-miR-18a	HMDD3.0, dbDEMC, PMID: 32146479
19	0.9505	hsa-let-7a	dbDEMC, PMID: 29398802
20	0.9489	hsa-miR-19a	HMDD3.0, dbDEMC, PMID: 34895042
21	0.9473	hsa-miR-222	HMDD3.0, dbDEMC, PMID: 20203269
22	0.9463	hsa-miR-143	dbDEMC, PMID: 28890884
23	0.9454	hsa-miR-31	HMDD3.0, dbDEMC, PMID: 22511990
24	0.9453	hsa-miR-29c	dbDEMC, PMID: 31333331
25	0.9445	hsa-miR-223	HMDD3.0, dbDEMC, PMID: 27900032
26	0.9443	hsa-miR-133a	dbDEMC, PMID: 32647415
27	0.9439	hsa-miR-199a	HMDD3.0, dbDEMC, PMID: 31636666
28	0.9409	hsa-let-7b	HMDD3.0, dbDEMC, PMID: 33283713
29	0.9398	hsa-miR-150	HMDD3.0, dbDEMC, PMID: 27917123
30	0.9386	hsa-miR-200b	PMID: 30574752

**Table 6:** The advantages and drawbacks of our method and other methods.

Method	AUC	Advantages	Drawbacks
PBMDA	0.9172	Topological information, complex network	No weighted, imbalance problem
WBNPMD	0.9173	Weighted edges	No topological information, imbalance problem
NIMCGCN	0.9291	Topological information, complex network, neural inductive	No weighted, imbalance problem
DNRLMF	0.9357	Complex network, dynamic regularized weight	No topological information, imbalance problem
VGAE-MDA	0.9394	Topological information, complex network, variational Bayesian inference	No weighted, imbalance problem
Ours	0.9472	Topological information, complex network, adaptive weight	Imbalance problem

**Table 7:** The framework and parameters of model.

	Parameters
GAT	Input (1, 857, 857)
	Node attention layer (1, 857, 32) × 8, activation function
	Concatenate layer (1, 857, 256)
	Module attention layer (857, 256), activation function
	Dense layer (857, 256), activation function
	Learning rate (0.001)
	Epoch (2000)
SVM	Kernel function (radial basis function)
	C factor (50)

## Data Availability

The datasets and codes are available at https://github.com/Excenmin/MMGAN-SVM (accessed on 1 March 2022).

## Sample Availability

Not available.
